# Supplementary material for: Splice-Junction-Based Mapping of Alternative Isoforms in the Human Proteome
Source: Cell Rep. Author manuscript; Available in PMC 2020 Jan 15. (PMC6961840; doi:10.1016/j.celrep.2019.11.026)
Supplement: 2 [file NIHMS1546469-supplement-2.zip › DF1/PXD006675/TricuspidValve/TricuspidValve_7_OBSCN_TVAELAVQAPR.pdf]

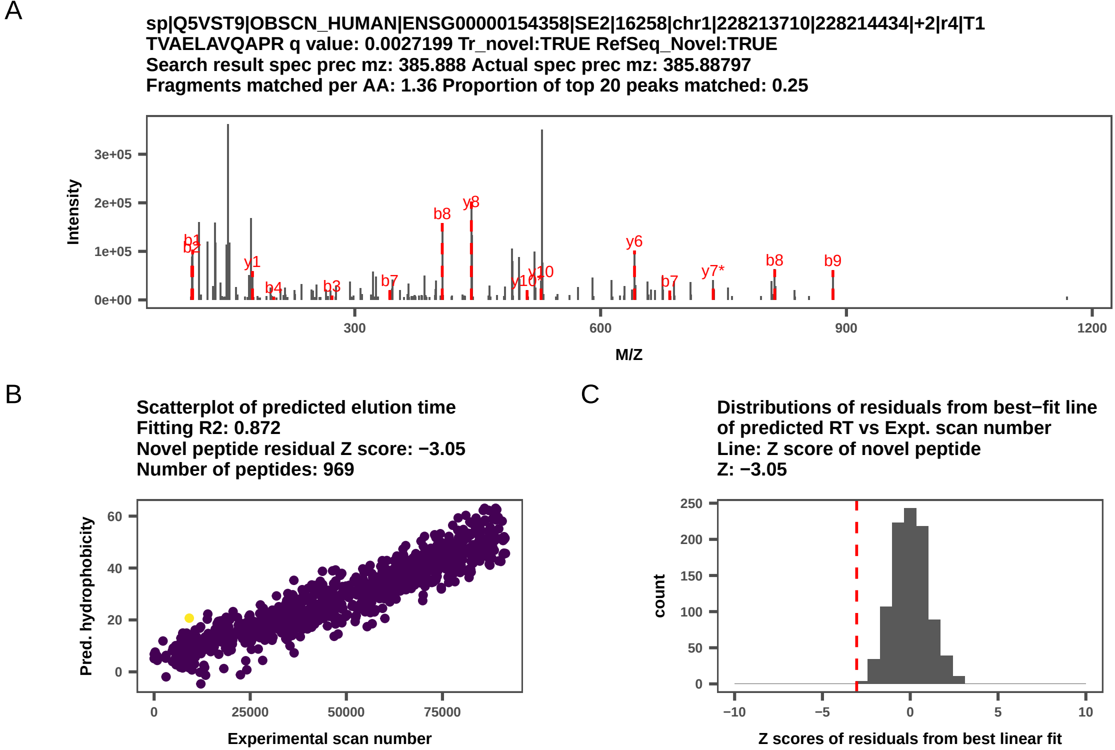
A
sp|Q5VST9|OBSCN_HUMAN|ENSG00000154358|SE2|16258|chr1|228213710|228214434|+2|r4|T1
TVAELAVQAPR q value: 0.0027199 Tr_novel:TRUE RefSeq_Novel:TRUE
Search result spec prec mz: 385.888 Actual spec prec mz: 385.88797
Fragments matched per AA: 1.36 Proportion of top 20 peaks matched: 0.25
Intensity
3e+05
2e+05
1e+05
0e+00
b1
b2
y1
b4
b3
b7
b8
y8
y10*
y10
y6
b7
y7*
b8
b9
300
600
900
1200
M/Z
B
Scatterplot of predicted elution time
Fitting R2: 0.872
Novel peptide residual Z score: −3.05
Number of peptides: 969
Pred. hydrophobicity
60
40
20
0
0
25000
50000
75000
Experimental scan number
C
Distributions of residuals from best−fit line
of predicted RT vs Expt. scan number
Line: Z score of novel peptide
Z: −3.05
count
250
200
150
100
50
0
−10
−5
0
5
10
Z scores of residuals from best linear fit
